# Formation of Olive-like TiO_2_ Nanospheres in a Polymeric Mesh by Sol-Gel Method

**DOI:** 10.3390/polym16131875

**Published:** 2024-06-30

**Authors:** Claudia López Melendez, Humberto Alejandro Monreal Romero, Caleb Carreño-Gallardo, Guillermo Martinez Mata, Rosaura Pacheco Santiesteban, Teresa Pérez Piñon, Dagoberto Pérez Piñon, Héctor Alfredo López Aguilar, Marvin Elco Estrada Macias, José Guadalupe Chacón-Nava

**Affiliations:** 1 Department of Engineering and Materials, La Salle University, Avenue Lómas de Majalca 1120, Chihuahua CP 31625, Chih., Mexico; clopez@ulsachihuahua.edu.mx (C.L.M.); hlopez@ulsachihuahua.edu.mx (H.A.L.A.); 2 Department of Biomaterials Science and Nanotechnology, University of Chihuahua (UACH), Avenue University, Chihuahua CP 31000, Chih., Mexico; gmata@uach.mx (G.M.M.); rpacheco@uach.mx (R.P.S.); mperez@uach.mx (T.P.P.); meestrada@uach.mx (M.E.E.M.); 3 Advanced Materials Research Center, S.C. (CIMAV) and National Nanotechnology Laboratory, Avenue M. Cervantes 120, Industrial Complex Chihuahua, Chihuahua CP 31136, Chih., Mexico; caleb.carreno@cimav.edu.mx (C.C.-G.); jose.chacon@cimav.edu.mx (J.G.C.-N.); 4 Department of Polymers and Biomaterials, University of Chihuahua (UACH), University Circuit Campus II, Chihuahua CP 31000, Chih., Mexico; dperezp@uach.mx

**Keywords:** titanium dioxide, olive-like TiO_2_ nanospheres, polysaccharide

## Abstract

Olive-like TiO_2_ (titanium dioxide), nanospheres compounds were synthesized. Polysaccharide (1–3 linked β-D galactapyranose and 1.4-linked 3.6 anyhdro-α-L-galactopyranose and titanium isopropoxide (IV) was used as a precursor in its formation. The powder sample was evaluated by scanning tunneling microscope, X-ray diffraction pattern, power spectral density, fast Fourier transform, differential thermal analysis, continuous wavelet transform, and isotropy texture analysis. The results demonstrate that these nanospheres can successfully be synthesized in a solution using a polysaccharide network by means of the sol-gel method. The synthesized olive-like TiO_2_ nanospheres have diameters ranging from 50 nm to 500 nm. The synthesis parameters, such as temperature, time, and concentration of the polysaccharide, were controlled in solution.

## 1. Introduction

The synthesis of materials using various organic compounds has increased today to study aspects related to molecular self-assembly to generate structures of diverse morphology, such as nanoparticles, coatings, and organic-based nanomaterials [1]. The formation of olive-type structures, with different properties, such as drug release, hydrogen storage reservoirs, and the use of organic molecules such as vitamins, amino acids, and proteins, has led to the exploration of new routes to its synthesis. The use of metal oxides such as titanium represents a favorable alternative for these activities due to their chemical stability within organic systems. It has been used in various areas, such as clinical medicine in the application of prostheses, alternative energy, and biosensors. Titanium has proven to be very versatile within these areas since its physical, chemical, and electronic properties can increase the kinetic reactions between the surface of the titanium and the molecules that interact in the aqueous medium. This immobilizes the ionic density in the molecular self-assembly processes, reducing fluid resistance when used as transport vehicles within different complex systems such as the cell, muscle fibers, and nanoelectromechanical systems. The elaboration of olive-type morphologies in the presence of polysaccharides can help bind titanium through electrostatic interactions, controlling the growth of particles along the three-dimensional network of the polysaccharide. Due to the presence of pores, nanospheres have good photocatalytic activity and have several applications, such as solar cells, wastewater treatment, pharmacology, and biomedical applications. Additionally, there are different methods in the preparation of nanospheres, such as arc discharge, solvothermal method, laser pyrolysis, directed assembly method, sol-gel method, and tempering method. Likewise, the different strategies of synthesis highlight the importance of the preparation of nanospheres for the formation of olive-like structures using different methods such as the electrodeposition technique, hydrothermal technique, glycolated precursors, and mini-emulsion processes [2,3,4,5]. One of the most interesting and versatile processes for this purpose is related to the use of TiO_2_ (titanium dioxide) with different polymeric precursors such as Polyvinyl alcohol (PVA), poly(ethyleneimine) (PEI), poly(sodium4-styrene sulfonate), polymethylmethacrylate, polyamide [6,7,8,9,10,11]. In this manner, the importance of the elaboration of new nanostructures allows the use of different techniques, such as the solvothermal method, green synthesis, and sol-gel [12,13,14]. Other techniques used to synthesize nanoporous nanostructured materials and nanoparticles is the use of polymeric membranes and cylindrical micelles, which exhibit special characteristics [15,16]. The synthesis of nanoparticles and nanospheres in the presence of polymeric networks plays an important role in the elaboration of various compounds that can be used in areas such as the pharmacology, tissue engineering, and ceramics industries [17,18,19]. Furthermore, a very interesting area in the development of different structures designed through organic molecules is the design of bioinspired materials using biological organisms, such as biopolymers, bone, and fibers [20]. These structures have interesting mechanical properties that make them suitable for the formation of spatial geometric figures, although they present some limitations due to their natural origin, such as being able to imitate their biomimetic properties [21]. Nevertheless, the goal is to combine the properties of each organic and inorganic material to obtain materials with specific properties, such as the use of repellent compounds in different solutions or materials with high mechanical strength [22]. In this manner, several strategies in the development of nanospheres can be engaged with the generation of bioinspired materials with various biological components, such as agarose, cellulose, and rhodamine [23,24,25]. Likewise, other types of materials have been used, such as SiO_2_, carbon, and CuCo2O_4_, for the synthesis of nanospheres using techniques such as protein separation, chemical vapor deposition, and heterogeneous contraction [26,27,28]. Many of these works attempt to explore molecular recognition processes to study chirality mechanisms when molecules of organic origin interact. Thus, studies with chiral polymers are highly promising for catalytic and optoelectronic activities. Furthermore, various nanostructures, such as nanoparticles, nanofibers, and nanospheres, can be explored using diverse technologies, such as scanning tunneling microscopy, scanning electron microscopy, and scanning probe microscopy. It is important to highlight the use of polysaccharides in the synthesis of nanospheres due to the multiple benefits of biocompatibility, non-toxicity, and ease of acquisition. In addition, polysaccharides have several physical, chemical, thermal, and optical characteristics that allow them to be used to generate a wide variety of nanoscale structures. Most of the physical, chemical, thermal, and optical processes are carried out at the nanometric scale, using a great diversity of biomolecules, inorganic molecules, and polypeptides. The resulting nanostructures, in some cases, are assembled to form complexes and materials with different geometric shapes. The forces involved are electrostatic, non-covalent interactions, ionic and hydrogen bonds, the hydrophobic and hydrophilic effect, and van der Waals forces. In this way, an important factor to consider regarding the olive-like TiO_2_ nanospheres synthesized with the polysaccharide network is that the interaction of hydrogen bonds, in aqueous means, improves the electrostatic bonding during the synthesis process. In this work, we have prepared olive-like TiO_2_ nanospheres, and the resulting nanospheres showed a heterogeneous size using the sol-gel method.

## 2. Materials and Methods

### 2.1. Synthesis and Characterization of Olive-like TiO_2_ Nanospheres

The TiO_2_ nanospheres were synthesized using the following methodology. The compounds were prepared with the addition of 1 g of polysaccharide (1–3-linked β-D galactapyranose and 1.4-linked 3.6 anyhdro-α-L-galactopyranose 99% Sigma-Aldrich St. Louis MO 63178, USA) as a template and 100 mL of bidistilled water in a solution of 300 mL of 99.8% ethanol (Sigma-Aldrich, St. Louis MO 63178 USA). Subsequently, the solution with a pH of 7.0 was subjected to a heating process at 50 °C for 5 min, 25 mL of titanium (IV) isopropoxide (Ti [OCH(CH3)2]4—97% (Sigma-Aldrich, St. Louis MO 63178 USA), 0.5 M, under constant stirring and drying at room temperature for gel formation for 8 min in the mold. In this way, the polymerization of the agarose occurs slowly, forming an intramolecular network controlling the growth of the spheres by minimizing the free energy caused by the surface tension of the polysaccharide fibers. Once the gel was formed, it was placed in ethanol, and the gel obtained was subjected to aging for 24 h. Afterward, the ethanol was exchanged 3 times over the course of 5 days, and the gel was heated to 500 °C for approximately 10 min. The heating purpose is to obtain the powders and establish the beginning of the transition of the crystalline phases of TiO_2_. Furthermore, it increases the density of TiO_2_ and slows down the degradation of the polysaccharide network without compromising the formation of the geometric shape of the nanospheres. It also preserves the cross-linking of their fibers.

The powders obtained were characterized with the following techniques.

### 2.2. Characterization of Olive-like TiO_2_ Nanospheres by FESEM

The olive-like TiO_2_ nanospheres were studied with a field emission scanning electron microscope (FESEM) JEOL-JCSM-7401F Peabody, MA 01960 USA. The specimens were placed in a holder with an acceleration voltage of 2.0 kV and a magnification of 30,000.

### 2.3. Characterization of Olive-like TiO_2_ Nanospheres by X-ray

For the characterization by X-ray diffraction, the samples of the powders began to be prepared and were placed in the sample holder, being careful to place them in the center and without the sample protruding from the walls of the latter so that the diffraction phenomenon occurred without problems, and the results were optimal for later study. The sample was placed in the first space of the diffractor, and it was verified that it was completely well placed so that the container or the sample would not fall. Next, the parameters in which the sample was to be studied were placed, as well as the place where the sample was located, and diffraction was carried out. The crystalline phase of the nanostructures was detected by an X-ray diffraction (XRD) pattern using a CuK (α) at 0.1542 nm on a Phillips X’. PERT Eindhoven AMS. X-ray diffractometer.

### 2.4. Characterization of Olive-like TiO_2_ Nanospheres by TGA-DTA-DTG Analysis

The TGA-DTA-DTG analysis of the nanospheres was processed on DTA-TGA TA, CDMX equipment at a heating rate of 10 °C/min in an air atmosphere.

### 2.5. Characterization of Olive-like TiO_2_ Nanospheres by STM

The characterization of the compounds was carried out through a Nanosurf easyScan 2, Liestal, Switzerland, instrument scanning tunneling microscope equipped with Pt/Ir tips (BT00400), and the images were processed using an easyScan 2 imaging software version 1-6-0-0 and a Mountains software version 9.0 United States Laboratory. The preparation of the samples for STM was carried out as follows: the powders were placed in 5 mL of bidistilled water and stirred in a Thermo scientific 16715 vortex to disperse the nanospheres; subsequently, the nanospheres were dried at room temperature for 24 h. Once dried, they were placed on a microscope glass slide-Esco of 1.2 mm thickness. The parameters used in STM were continuous mode at 20 nN, the tip voltage was 1 V, the sweep speed was 0.1 s, and the operating mode was static force. The image resolution area was 161 × 161 nm. 

### 2.6. Characterization of Olive-like TiO_2_ Nanospheres by Mountains Lab Software

The analysis of power spectral density (PSD), fast Fourier transform (FFT), surface geometry analysis, fractal dimension analysis, coefficient of determination R2, continuous wavelet transform analysis, decomposition of the continuous signal, profile of holes analysis, and texture isotropy analysis was executed utilizing the Mountains Lab 9.0 image processing software. The images obtained through STM and FESEM were processed in this software using the following parameters: structural analysis, texture direction, control chart, and critical dimension analysis.

## 3. Results and Discussion

### 3.1. Characterization of Olive-like TiO_2_ Nanospheres by SEM

**Figure 1:**
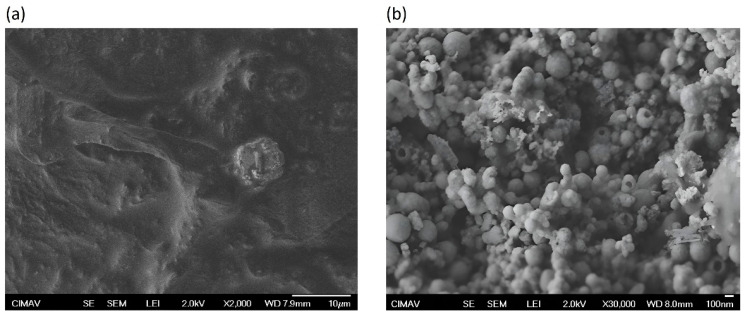
(**a**) SEM image of TiO_2_ (titanium dioxide) in absence of polysaccharide; (**b**) SEM micrograph of olive-like TiO_2_ nanospheres in presence of polysaccharide network.

Figure 1a shows the SEM micrograph of TiO_2_ in the absence of the polysaccharide matrix, it can be seen that there are no nanospheres present, and the surface is irregular. Figure 1b shows the image in SEM of the olive-like TiO_2_ nanospheres in the presence of a polysaccharide matrix with diameters of 50–500 nm. This type of morphology offers the advantage of being used as a vehicle to store different substances, such as drugs, proteins, and electronic nanodevices, compared to those existing in the literature. TiO_2_ nanospheres have been reported with sizes between 56 and 100 nm using polysaccharides such as agarose and alginate [23,24,25,26,27,28,29]. The image presents heterogeneous morphological characteristics, and the hole of the nanospheres is probably due to the polysaccharide matrix. Also, these images suggest that the electrical behavior of the electrostatic junction composed of polysaccharide and TiO_2_ is controlled by an electron transfer mechanism characteristic of molecular self-assembly systems in solution. Molecular self-assembly is a process of spontaneous organization with electrostatic interactions under several conditions of equilibrium, such as hydrogen bonds of the polysaccharides, non-covalent bonds, hydrophobic and hydrophilic interactions, and polymerization of processes.

### 3.2. Particle Size Distribution

**Figure 2:**
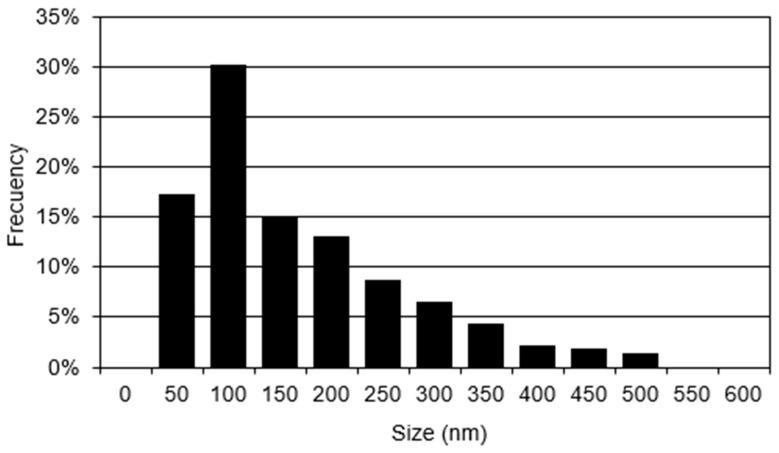
Chart of particle size distribution of olive-like TiO_2_ nanospheres.

Figure 2 shows the particle size distribution with ranges of 50–500 nm. Approximately 30% of the nanospheres obtained are in the range of about 100 nm in diameter. In another study, a particle size in the range of 100–200 nm was reported using the biphasic system [30]. Moreover, the average particle size, utilizing the polymeric system, permits the biological activity to be fixed on the surface of the nanospheres or in the aqueous medium. In other words, the control of the properties of the linear polymer and the particle size determine the characteristics of each sphere.

### 3.3. X-ray Diffraction Pattern

**Figure 3:**
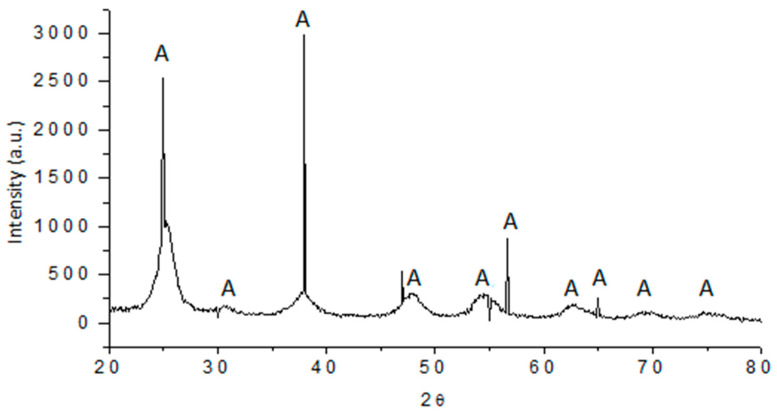
X-ray diffraction pattern of olive-like TiO_2_ nanospheres.

The crystalline phase of the olive-like TiO_2_ nanospheres was identified by X-ray diffraction patterns at 500 °C, Figure 3. In these patterns, the reflections at 2θ of 25°, 31°, 38°, 48°, 55°, 57°, 63°, 65°, 70°, and 75° visibly correspond to the anatase phase of TiO_2_. The system used allowed the obtaining of crystalline nanospheres, avoiding subsequent sintering methods that could induce TiO_2_ to crystallize, affecting the ability to adsorb the polysaccharide network. These results are similar to those found in other works during the synthesis of TiO_2_ nanospheres [31,32,33]. The degree of the crystalline phase is approximately 98%, and the intensity of the reflections of the anatase phase can be observed. A separation of the amorphous phase can be observed before the first reflection, and later, the other reflections appear, which remain during the sintering process. 

### 3.4. Thermo-Gravimetric and Differential Thermal Analysis

**Figure 4:**
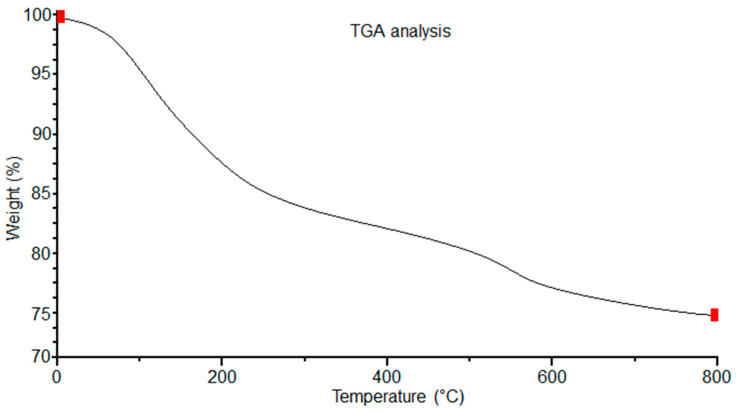
TGA analysis of the olive-like TiO_2_ nanospheres.

**Figure 5:**
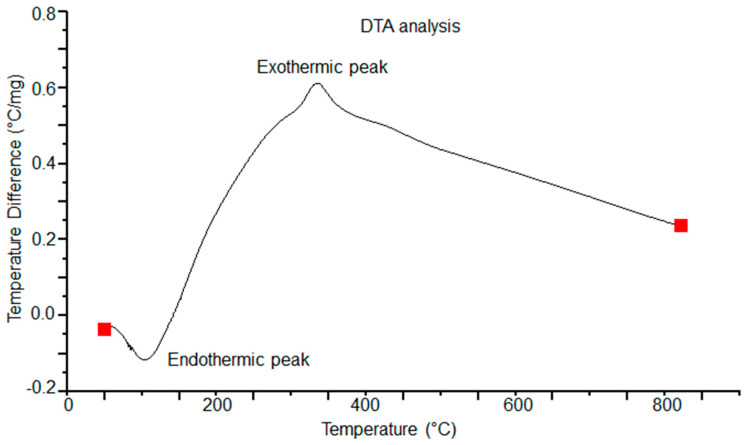
DTA analysis of the olive-like TiO_2_ nanospheres.

In Figure 4 and Figure 5, the results of the TGA-DTA study of the olive-like TiO_2_ nanospheres are shown. The temperature region of the TGA curve, Figure 4, shows a weight loss at 100–200 °C. This behavior corresponds to the loss of water and alcohol that causes the appearance of an endothermic peak at 60 °C and an exothermic peak at 300 °C, Figure 5, caused by the combustion of organic matter and the titanium precursor. The polysaccharide fibers have oxidation degrees of the residual galactose and agarobiose groups, which means that there is no loss of weight at 45 °C. This behavior is due to a process of direct coagulation or chemical gelification of the polysaccharide, which allows it to increase the density of TiO_2_ and decrease the fast degradation of the polysaccharide.

**Figure 6:**
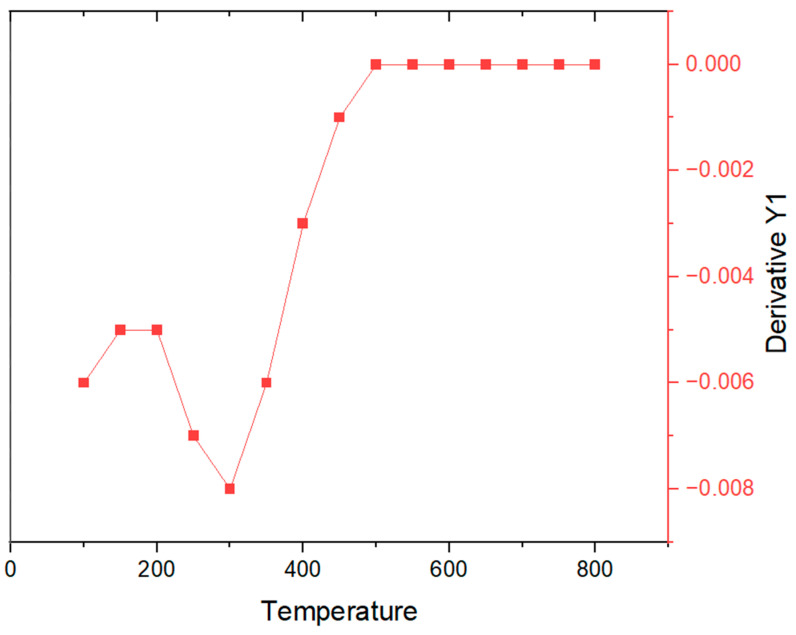
DTG analysis of the olive-like TiO_2_ nanospheres.

In addition, there is a slow and continuous degradation of the polysaccharide matrix at temperatures between 65 and 600 °C. The permanence and evolution of the anatase phase is at approximately 500 °C. This prevalence may be due to the kinetic processes of phase transformation, in which the polysaccharide matrix is capable of retaining the anatase phase, inhibiting the nucleation processes for the formation of the rutile phase. Figure 6 shows the DTG analysis of the nanospheres. In the first, second, and third points of the curve, the mass loss occurs in a slow process between 100 and 200 °C. This loss may be due to the desorption of water adsorbed on the surface of the nanospheres. A fourth point is seen at approximately 250 °C; according to this, as the temperature increases, the loss of mass becomes even slower, reaching a curve at 300 °C. We report that the total weight loss is 22.5%. Subsequently, the stabilization of the curve begins at approximately 500 °C. The observed behavior may be due to the breaking of chemical bonds, causing the three-dimensional network of the polysaccharide to decompose and resulting in the thermal degradation of TiO_2_. In this way, the presence of these curves may represent the stability of TiO_2_ in relation to the three-dimensional network of the polysaccharide carried out by a complex thermal degradation interaction.

### 3.5. Characterization of Olive-like TiO_2_ Nanospheres by Scanning Tunneling Microscopy (STM)

**Figure 7:**
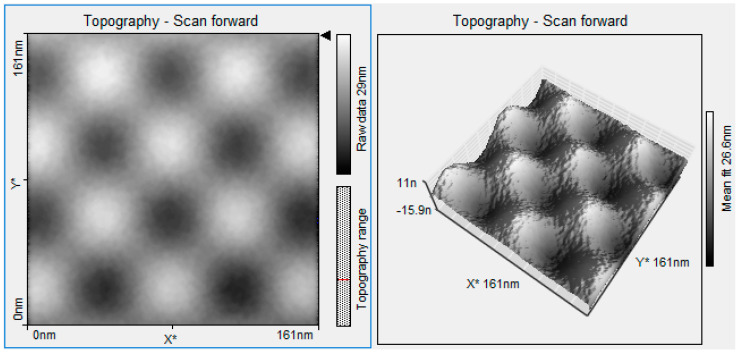
STM image of olive-like TiO_2_ nanospheres.

Figure 7 shows a complex of nanospheres as part of the integration into a polysaccharide matrix, thereby demonstrating that compatibility exists and electronic activity is prevalent. The observed behavior of nanospheres at low temperatures in STM suggests, however, that the interaction of the adatom would be located in the central region between the rest of the atoms, as shown in Figure 7. In this manner, for the various partially occupied orbitals that are present in the unit cell of the reconstruction of Ti (110), an element more electronegative than the surface (electron acceptor) would have a preference to interact with the polysaccharide. Therefore, if there is an interaction with an electron acceptor component (more electronegative than the surface), there will be a charge transfer from the surface to the element. In both cases, there is a flow of electrons characterized by a variation in the chemical potential of electrons. This will have a model system to transfer (donate or accept) electrons. The study also suggests that the increase in the function of the polysaccharide matrix might be due to the similarity of the fibers and the TiO_2_ crystals found in the nanospheres. The binding of Ti-O-H molecules within a solution, such as ethanol, increases the additional energy of the Fermi level, causing the nanospheres to increase electrical conductivity. The difficulty of performing surface topography analysis on monosaccharides derives from the electrostatic charges emitted by STM. These results demonstrate the self-assembly of the nanospheres in the polysaccharide matrix, reducing the generation of unnecessary electrical charges and, thus, allowing the effective union of oxygen with Ti. In this way, the electronegative OR group of the alkoxide renders the metal very susceptible to nucleophilic attack, leaving a partial negative charge. This, during the reaction, allows the polysaccharide to provide a system capable of forming hydrogen bonds and reacting with the hydrolyzed Ti-OH species. During the polymerization process, in the presence of the polysaccharide network, the Ti-OR is converted into a Ti-OH bond. The results also demonstrate that the nanospheres induce a fusion process within the solution by modifying the supramolecular structure of the interpenetrated networks of the polysaccharide. Nanospheres allow the miniaturization and functionality of the networks, allowing the force fields between molecules to be optimal for interaction. This characteristic is what allows, through electrostatic interactions, in an aqueous medium, for the compounds in the solution, mainly the precursors of the titanium and water used, and the polysaccharide fibers, to interact, finally forming the structures and giving rise to the chemical modification of the surface results, favoring adhesion to the TiO_2_ surface. Several studies have been developed in which the properties of metal oxides such as TiO_2_ and the activity of oxygen vacancies in nanospheres have been analyzed, but this activity has not been reported with polysaccharides such as those performed in this study [34,35,36].

### 3.6. Characterization of Olive-like TiO_2_ Nanospheres by FFT

**Figure 8:**
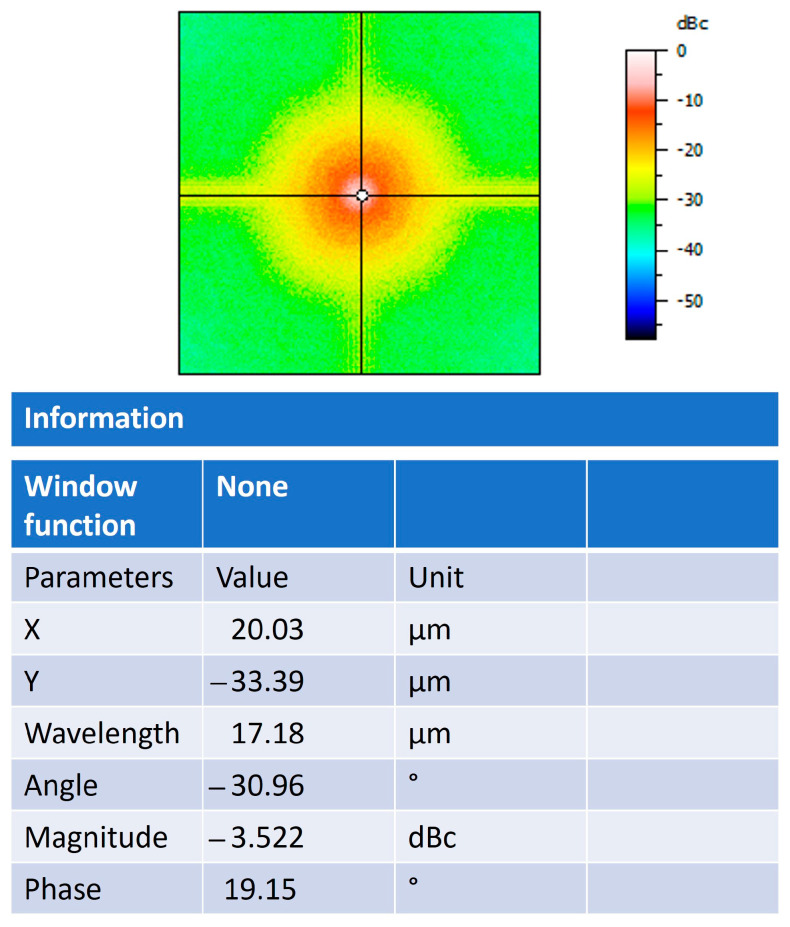
The frequency spectrum of FFT.

The frequency spectrum of the fast Fourier transform is observed in Figure 8; the nanospheres produce high frequencies in their intensity levels, which are observed as parts with greater luminosity due to the presence of TiO_2_. They can also cause low frequencies that are shown as less intense parts that are caused by the presence of the polysaccharide matrix. The value of power relative to carrier (dBc) analysis of −3522 indicates that the nanospheres have a higher power in the carrier signal than in the power of the input signal. In this way, greater power allows greater interaction in the molecular self-assembly process, leading to a stronger union. In this way, when the aggregates in each pore of the polysaccharide network align to form the nanospheres, there is an unequal number of negatively charged monomers in each pore, resulting in a molecular dipole throughout the matrix. Depending on the orientation of this molecular dipole, an applied voltage can mobilize and destabilize the aggregates, providing voltage-dependent ionic transport. In this manner, the amplitude of the fast Fourier transform is taken by the power spectral density to normalize the frequency width.

### 3.7. Characterization by Power Spectral Density (PSD) Analysis of Olive-like TiO_2_ Nanospheres

**Figure 9:**
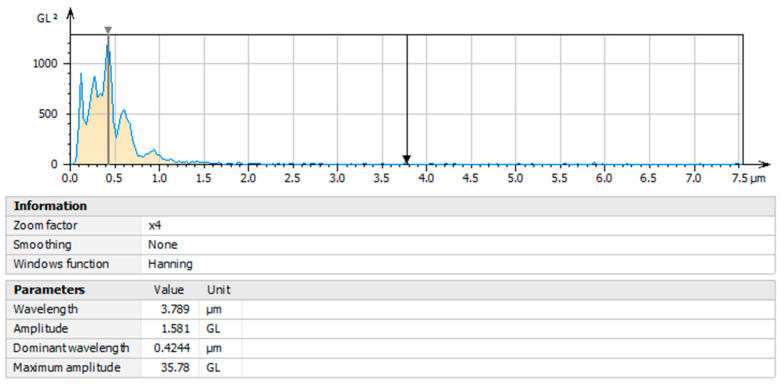
Power spectral density (PSD) analysis.

The image of Figure 9 shows the characterization of the nanospheres by means of the force spectral density analysis; the area of the square of the curve that corresponds to the energy of the signal, both vertical and lateral, is shown. The results are distinctive in representing the function of the moisture absorption capacity and the property of the nanospheres to scatter light emissions. The dominant energy corresponding to the wavelength presents a maximum peak of 0.4244 µm with an amplitude of 1.581 gL; the graph shows a decrease in its curve with the presence of two peaks, one at 0.6 µm and the other at 0.9 µm. The interaction of TiO_2_, ethanol, and polysaccharide facilitates the appearance of peaks that contain force periodicity signals, controlling the existence of electron vibrations and canceling the presence of noise during the temperature application process in the gels. In this context, the level of polarization may depend on the electromagnetic power of the nanospheres and the dipole moment at high and low frequencies, with changes in the dielectric balance.

### 3.8. Fractal Dimension Analysis

**Figure 10:**
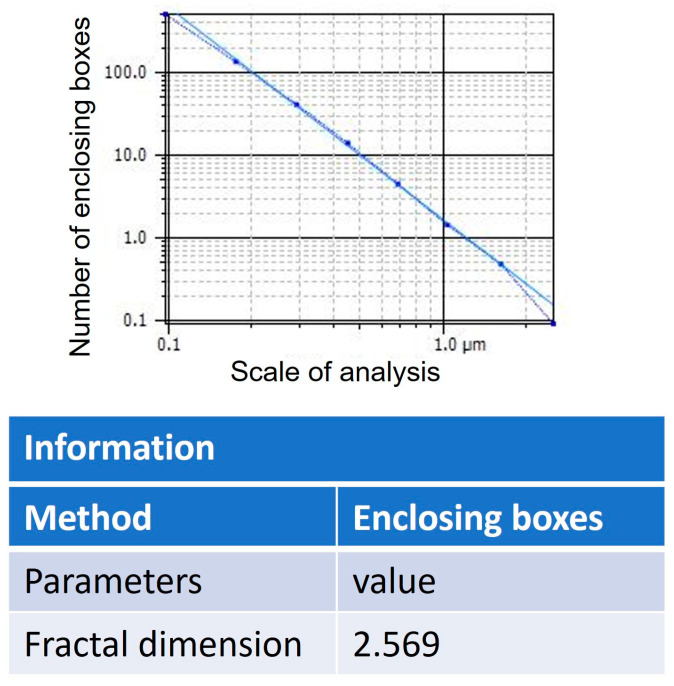
Fractal dimension analysis of olive-like TiO_2_ nanospheres.

**Figure 11:**
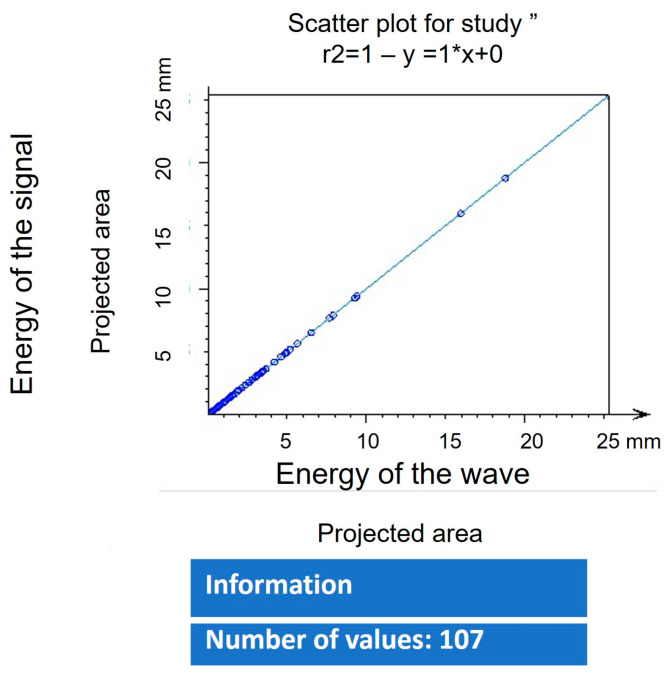
Coefficient of determination R2.

The values of the analysis of the fractal dimension are shown in Figure 10. The olive-like TiO_2_ nanospheres were analyzed using the following equation:

(1) $$\log S=D\log L=D=\log\frac{S}{\log L}$$

where *S* (8) is the size of the fractal, *L* is the measurement scale (2.246), and *D* is the unknown fractal dimension. The nanospheres present a homogeneous morphology, and the values of the fractal dimension of 2.569, as well as the R2 correlation data, establish the existence of a positive relationship with a value of 1, which demonstrates that the nanosphere formation system can be 100% replicated; see Figure 11. In this way, the system evolves and organizes into a state or structure that is consistent with the decrease in the total free energy of the system, including the free energies present in the volume as well as the free energies present at the interface. The energies can be elastic strain energies due to the mismatch between the various phases as well as between the real system and the substrate. The interface energy leads to the selection of those surfaces and interfaces that have the lowest energies. Kinematically controlled nanospheres are governed by variables such as deposition, time, and diffusion fluxes from the metal surface. The structure that is being formed is the result of the interaction of these processes, which act simultaneously during the deposition process of the metal precursor. Finally, depending on the different precursors, the growth rate is minimal, even in the presence of other acidic or basic media. Furthermore, the growth rate also depends on the concentration of the dispersing medium. Likewise, the result of the fractal dimension is related to the morphology of the edges since it presents an irregular surface that is continuous and characteristic of its geometric shape, which is due to the participation of the polysaccharide matrix. The study of particle addition and the formation of fractals in TiO_2_ nanostructures have been reported in several works [37,38,39,40,41].

### 3.9. Characterization by Continuous Wavelet Transform (CWT)

**Figure 12:**
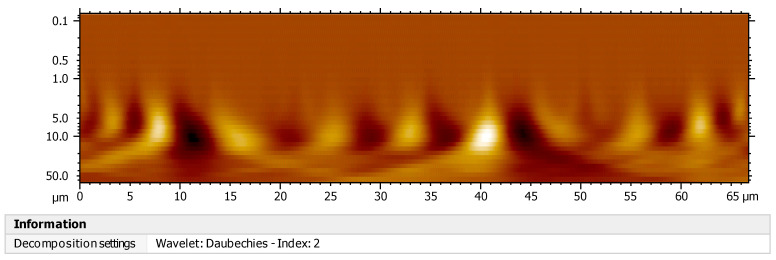
Wavelets of olive-like TiO_2_ nanospheres.

The continuous wavelet transform was used for the determination of the origin of the wave propagation of the mother signal in the composite materials of polysaccharide and Ti, and the textural characterization of the nanosphere surface to detect anomalies under the presence of wavelets. Figure 12 shows orthonormal wavelets, which means that they meet the orthonormality conditions within the same signal scale. The *x*-axis represents the position along the signal (time), the *y*-axis represents the scale, and the color represents the magnitude of the wave coefficient. The continuous wavelet transform is determined by the following equation:

(2) $$Xwt\left(a,b\right)=⨜\infty x\left(t\right)\Psi a,b\left(t\right)dt$$

**Figure 13:**
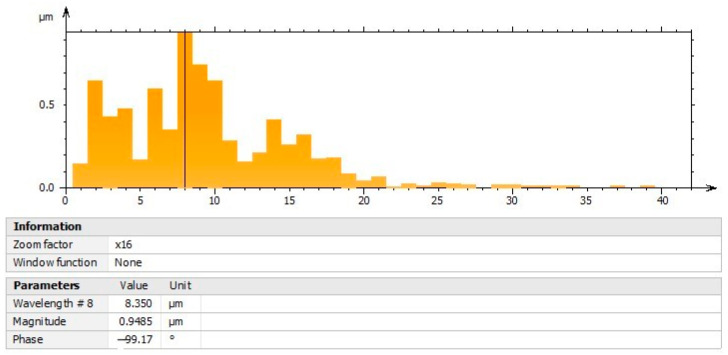
Histogram of signal decomposition of olive-like TiO_2_ nanospheres.

The mother wavelet generates several daughter wavelets that are added to the time of the signal *f*(*t*), originating sinusoidal components of the original signal. The image shows many of the wavelet coefficients (front and back), which are related to the scale and position of the mother wavelet. The peaks found in the wavelets can be caused by the effect of temperature at the time of sintering, which generates wear in the polymer chains. Figure 13 shows the histogram of the decomposition of the continuous signal. The signal values are greater for a scale factor of 8, 9, and 10 µm, which means that with that scale and at those positions, the similarity in the signals is greater, denoting that the energy of the signal and the energy of the wave are equal to one; this can be interpreted as a correlation coefficient (Figure 11). Likewise, the proposed system originates high- and low-frequency signals that vary in the time–frequency plane, causing high and low temporal resolutions, resulting in no discontinuity on the surface of the nanospheres. The columns of Figure 13 indicate non-periodic signal coefficients (wavelets) with short-duration pulses that make it possible to rapidly detect molecular self-assembly events between the compounds. The results do not show the presence of irregular edges on the surface; these data are conclusive in their determination of great compatibility between the interpenetrated networks of the polysaccharide with titanium. Other studies have analyzed the behavior of dispersive waves and frequency timing by means of cwt in TiO_2_ films, circular structures, and other nanostructures [42,43,44].

### 3.10. Characterization of the Profile of the Holes of Olive-like TiO_2_ Nanospheres

**Figure 14:**
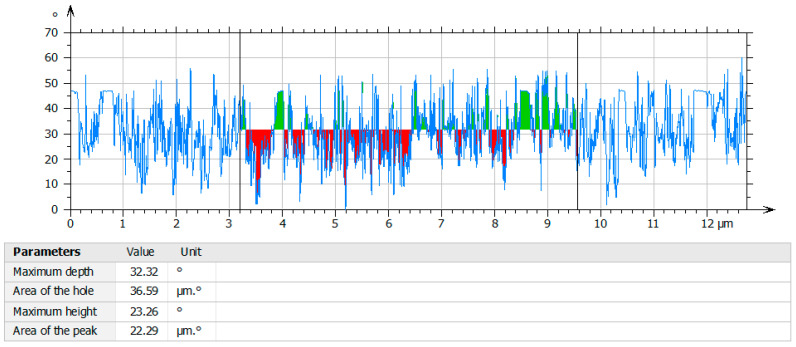
Profile of holes of olive-like TiO_2_ nanospheres.

The profile of the holes in the center of the nanospheres is shown in Figure 14. The holes are represented in red, while the green color corresponds to the titanium precursor. The formation of the holes takes place by means of the polysaccharide host molecule, which has cavities that can retain the TiO_2_. The nanospheres have a maximum depth of 32.32 gL and an area of 36.59 µm gL. In this way, the proposed system generates supramolecular architectures from metallic TiO_2_ ions and organic ligands such as polysaccharide polymer networks. In a study developed by Nora et al., the shell thickness of the surface of the TiO_2_ nanospheres was analyzed; however, their depth and area were not measured [45].

### 3.11. Texture Isotropy Analysis

**Figure 15:**
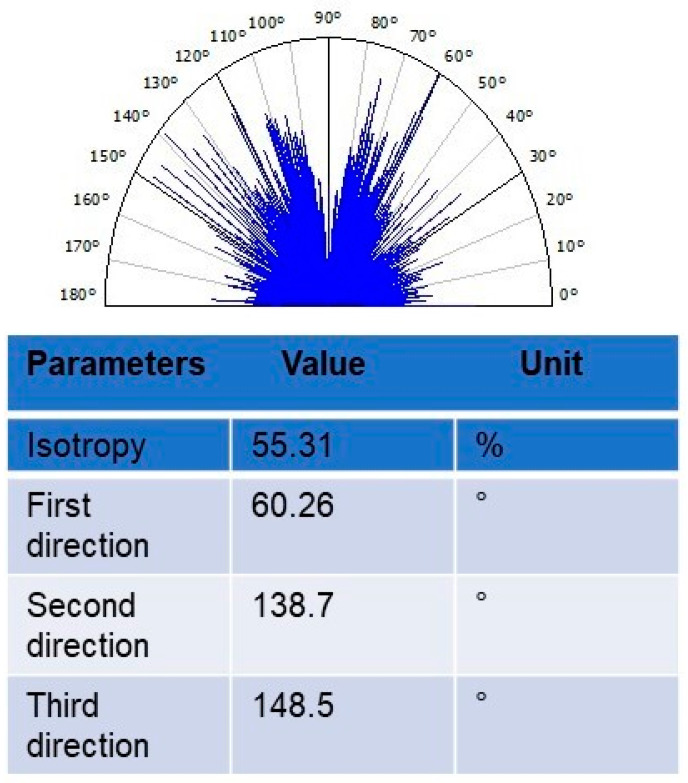
Texture isotropy analysis of olive-like TiO_2_ nanospheres.

To measure the directional uniformity parameters of the surface planes of the olive-like TiO_2_ nanospheres, a textural isotropy analysis was performed. The characteristics of the direction of the surface texture can be seen in Figure 15. The results of the values found were isotropy 55.31%, first direction 60.26°, second direction 138.7°, and third direction 148.5°, respectively. These results show the isotropic nature of the system, maintaining the uniformity of the surface features in all directions. Various structures, such as Cu/Ni nanoparticles, collagen fibers, cells, and carbon nanotubes, have been analyzed, but systems such as the one proposed in this work have not been studied [46,47,48,49,50]. Additionally, the location of each point on the surface of the sphere is represented by the angular degrees corresponding to the specific rotation coordinates of the number of grains in the sample in several directions. This means that the diverse molecules that contain the nanospheres are evenly distributed, which corresponds to the isotropic texture of the olive-type TiO_2_ nanospheres. 

## 4. Conclusions

In this work, we have reported the formation of olive-like TiO_2_ nanospheres in a range of 50–500 nm in diameter. The compounds synthesized can be replicated in other areas, such as biomaterials, nanotechnology, or materials science. The crystallinity of the nanospheres calcined at 500 °C is confirmed by X-ray. In summary, the olive-like TiO_2_ nanospheres were examined to determine their effects on the viability and adhesion of the polysaccharide matrix. Additionally, the proposed mechanism and the characteristics mentioned are factors of great relevance to studying the effects of the polysaccharide network and to be able to analyze variables, such as thermodynamic activity, electrostatic interactions, and organic ligands. The fractal dimension values with a value of 2.569 found in the fractal dimension analysis highlight the binding between the titanium and the polysaccharides network system. The functional activity of olive-like TiO_2_ nanospheres was confirmed by STM and FESEM in terms of morphological and size characteristics. Other studies of interest include corrosion studies and biocompatibility studies of metallic oxides.

## Data Availability

Data are contained within the article. For more information can be directed to the corresponding author.
